# Discriminating the earliest stages of mammary carcinoma using myoepithelial and proliferative markers

**DOI:** 10.1371/journal.pone.0201370

**Published:** 2018-07-25

**Authors:** Hendrika M. Duivenvoorden, Alex Spurling, Sandra A. O’Toole, Belinda S. Parker

**Affiliations:** 1 Department of Biochemistry and Genetics, La Trobe Institute for Molecular Science, Melbourne, VIC, Australia; 2 Sydney Medical School, University of Sydney, Camperdown, NSW, Australia; 3 Garvan Institute of Medical Research, Darlinghurst, NSW, Australia; 4 Australian Clinical Labs, Bella Vista, NSW, Australia; University of Texas Health Science Center, UNITED STATES

## Abstract

Mammographic screening has led to increased detection of breast cancer at a pre-invasive state, hence modelling the earliest stages of breast cancer invasion is important in defining candidate biomarkers to predict risk of relapse. Discrimination of pre-invasive from invasive lesions is critically important for such studies. Myoepithelial cells are the barrier between epithelial cells and the surrounding stroma in the breast ductal system. A number of myoepithelial immunohistochemistry markers have been identified and validated in human tissue for use by pathologists as diagnostic tools to distinguish *in situ* carcinoma from invasive breast cancer. However, robust myoepithelial markers for mouse mammary tissue have been largely under-utilised. Here, we investigated the utility of the myoepithelial markers smooth muscle actin (SMA), smooth muscle myosin heavy chain (SMMHC), cytokeratin-14 (CK14) and p63 to discriminate mammary intraepithelial neoplasia (MIN) from invasive disease in the C57BL/6J MMTV-PyMT transgenic model of mammary carcinoma. We identified that SMMHC and CK14 are retained in early *in situ* neoplasia and are appropriate markers for distinguishing MIN from invasive disease in this model. Additionally, the proliferation marker Ki67 is a superior marker for differentiating between normal and hyperplastic ducts, prior to the development of MIN. Based on this, we developed a scoring matrix for discriminating normal, hyperplasia, MIN and invasive lesions in this spontaneous mammary tumorigenesis model. This study demonstrates heterogeneous expression of myoepithelial proteins throughout tumour development, and highlights the need to characterise the most appropriate markers in other models of early breast cancer to allow accurate classification of disease state.

## Introduction

Increasing numbers of women (~25%) are now diagnosed with breast cancer at the pre-invasive state, ductal carcinoma *in situ* (DCIS) (reviewed in [1]) due to mammographic screening. DCIS is a state in which malignant epithelial cells are confined to the ducts and are surrounded by a boundary of myoepithelial cells [2]. Myoepithelial cells form a continuous single layer aligned parallel to the ducts between the inner layer of luminal epithelial cells and the surrounding stroma and secrete the basement membrane [3–5]. The presence of an intact myoepithelial cell layer is characteristic of DCIS, however disruption of the myoepithelial boundary can lead to invasive carcinoma [6]. It is thought that myoepithelial cells form a physical barrier which tumour cells must transverse in order to become invasive, and there is evidence to support that the transition from DCIS to invasive carcinoma is regulated by the loss of normally functioning myoepithelial cells [7]. Undoubtedly, myoepithelial cells have important roles in tumour suppression. Here we focus predominantly on their presence or absence for identifying tumour stage.

Pathologists use the presence of an intact myoepithelial layer to assist in distinguishing between DCIS and invasive carcinoma. A number of immunohistochemical markers have been used to differentiate myoepithelial cells from luminal epithelial cells in the breast, including α-smooth muscle actin (SMA), smooth muscle myosin heavy chain (SMMHC), p63, h-caldesmon, maspin, S100 protein, P-cadherin, cytokeratin (CK)-5, CK-14 and CK-17 [8]. Pathologists may use any combination of these to determine if a lesion is DCIS or invasive, however, some of these markers are less sensitive and are reported to be lost in some DCIS-associated myoepithelial cells [9–11]. Therefore, a combination of SMMHC and p63 are commonly used in routine practice.

As there is currently no accurate way to predict the development of invasive disease after a diagnosis of DCIS, identifying molecular drivers or suppressors of invasion is important and may lead to the discovery of prognostic biomarkers to predict risk of invasive disease and to inform patient treatment decisions [12–15]. Assessment of early invasion requires modelling the earliest stages of tumorigenesis [16, 17]. While there are *in vivo* models available, myoepithelial markers used to distinguish DCIS from invasion have not been well characterised in transgenic models of mammary tumorigenesis. This is a significant weakness for conducting early breast cancer studies *in vivo*, where discrimination of intraepithelial neoplasia from the earliest stages of invasive carcinoma is essential but may be challenging based on histological appearances alone. Mice are used as a model organism for researching mammary architecture and tumour development [18]. Transgenic models of spontaneous mammary cancer initiation are particularly useful for modeling the earliest stages of tumour invasion and progression, stages that are not represented in orthotopic transplant models.

One model that reproducibly recapitulates spontaneous mammary cancer is the mouse mammary tumour virus–polyoma virus middle T antigen (MMTV-PyMT) transgenic mouse model [19, 17]. Polyoma Virus middle T antigen (PyMT) is a protein encoded by the polyoma virus that is capable of establishing and maintaining transformed cells [20]. The MMTV long terminal repeat promoter confers specific expression of PyMT in mammary gland epithelium [21] whereby multifocal adenocarcinomas of the mammary gland develop at high penetrance as early as 3 weeks of age, with lung metastases detectable in 15–90% of mice by 9–16 weeks of age, respectively, depending on the strain [21, 19]. The PyMT mammary tumours progress with some morphological similarities to human cancer pathology, along with a similar pattern of expression of breast cancer biomarkers (such as loss of estrogen and progesterone receptors, and overexpression of cyclin D1 and erbB2/neu), making it a robust model to study tumour progression and microenvironment [14]. The C57BL/6J PyMT model progresses through hyperplasia, mammary intraepithelial neoplasia (MIN, resembling human DCIS), early and invasive carcinoma. Our recent studies using this model revealed that the MIN lesions had reduced expression of myoepithelial markers compared to human tissue [22], making it difficult to distinguish MIN from invasive carcinoma. The lack of standardised, diagnostic criteria to define each type of lesion for mouse tissue represents a gap in the field whereby discrimination of such lesions is difficult without an expert pathologist. Here, we measured alterations in myoepithelial and proliferation markers throughout defined stages of early tumorigenesis. Based on the findings herein, we have developed a scoring matrix for discriminating normal, hyperplasia, MIN and invasive carcinoma in the C57BL/6J MMTV-PyMT model of tumorigenesis as a tool for other researchers.

## Materials and methods

### Mouse models/histology

Mouse investigations were performed after approval by the La Trobe University Animal Ethics Committee. Mice were housed in a 501cm^2^ floor area cage, with 12 hour light/dark cycling and unrestricted access to food and water. Mice were euthanized by CO_2_ inhalation and/or cervical dislocation. Mammary glands from virgin female Balb/c wild type (50 days), C57BL/6J wild type (50 days) and C57BL/6J MMTV-PyMT positive mice [23] (20–70 days) were dissected, formalin fixed and paraffin embedded. At a minimum, three mice per time point were used.

For human tissues, samples from reduction mammoplasty or breast cancer patients were obtained from the Royal Prince Alfred Hospital (RPAH) and supplied as de-identified formalin fixed, paraffin embedded sections. Ethical approval for a waiver of consent for the use of archived, retrospectively collected human tissues was approved by the HREC of RPAH (approval number X15-0388 [SSA/16/RPAH/397]).

Haematoxylin and eosin (H&E) staining was conducted following standard protocols.

### Immunohistochemistry (IHC)

Fresh sections (formalin-fixed, paraffin embedded) were dewaxed following standard protocols and antigen retrieval with pH6 citrate retrieval buffer was undertaken for SMMHC, CK14, p63 and Ki67 staining. Sections were stained with anti-α-smooth muscle actin (1 μg/ml, Abcam, ab66133), anti-smooth muscle myosin heavy chain 1+2 (1 μg/ml, Abcam, ab124679), anti-Ki67 (1 μg/ml, Abcam, ab15580), anti-cytokeratin 14 (5 μg/ml, Abcam, ab53115), anti-p63 (0.75 μg/ml, Abcam, ab124762), or with isotype control antibodies, overnight at 4°C. Biotin-conjugated secondary antibodies (Vector Laboratories, CA, USA) were applied for 1 h and signal was amplified by conjugation of HRP (ABC kit, Vector Laboratories). Antibodies were visualized with DAB prior to nuclear counterstaining with hematoxylin.

### Statistical analysis

Statistics were conducted using the data analysis software package within GraphPad Prism v7 for Windows (GraphPad Software, La Jolla, CA, USA) including one-way ANOVA, Fisher’s exact test, mean and 95% confidence interval. Error bars indicate SEM unless otherwise stated.

## Results

### Early tumour progression in the C57BL/6J MMTV-PyMT model

**Fig 1:**
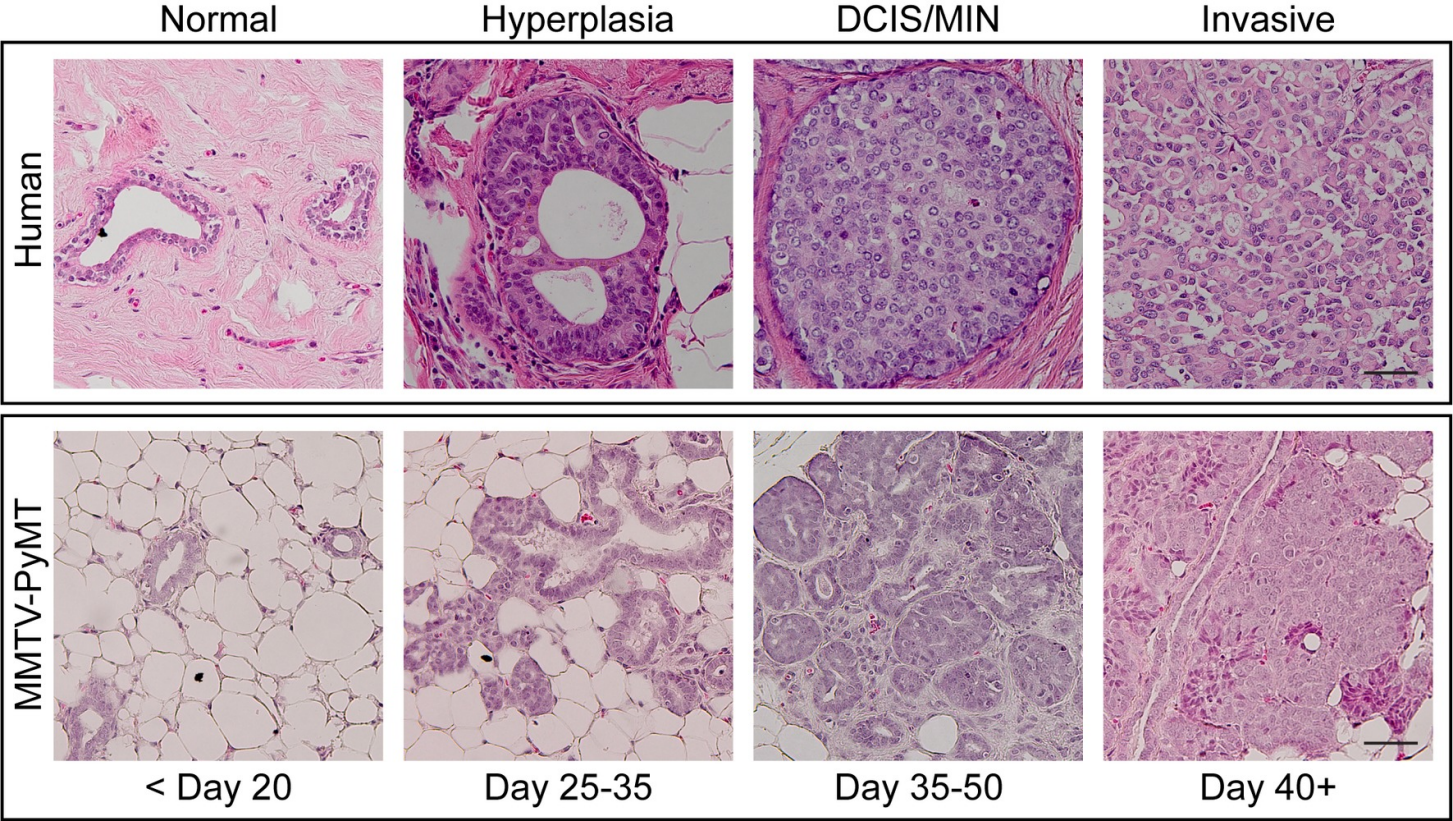
Penetrance of tumorigenesis in C57Bl/6J MMTV-PyMT mouse model compared to human disease. Sections of breast tissue from humans and mammary glands from C57BL/6J MMTV-PyMT mice at different ages classed as normal, hyperplasia, DCIS/MIN or invasive were stained by H&E. Scale bars represent 50 μm. Representative of >3 mice.

To confirm the timing of spontaneous development of mammary gland tumours in the C57BL/6J MMTV-PyMT model, mammary glands of C57BL/6J MMTV-PyMT mice at different stages were taken and morphologically evaluated by H&E (Fig 1) by an expert human breast and murine mammary gland pathologist (O’Toole). The microanatomy of the tissues at different tumour stages were compared with that seen in human breast cancers (Fig 1). In C57BL/6J MMTV-PyMT positive mice, hyperplasia was observed at approximately day 30, which correlates with previous findings of hyperplasia development at approximately 28 days in this strain of mice [19]. The morphology of hyperplastic lesions varies from that seen in patient-derived tissues (Fig 1). Hyperplasia in the mouse appears as a proliferation of glands/lobules localised in one area, with many ducts visible within one field (Fig 1, 200x magnification), and a single layer of luminal epithelial cells surrounding the lumen [14]. In contrast, usual ductal hyperplasia (UDH) in human tissue is characterised by a solid or fenestrated proliferation of cells in the lumen of duct spaces showing a typically syncytial or streaming pattern with slit-like secondary lumina often present, with only one-two ducts visible per field (Fig 1) [11]. Additionally, the cells of human UDH often have indistinct borders and variably sized nuclei with haphazard orientation in regard to one another [11]. Both MIN and invasive carcinoma were detected by 50 days of age in the MMTV-PyMT model (Fig 1). In our experience with this mouse strain, if not taken at these early ages for analysis of hyperplastic and MIN lesions, over 95% of the mice will develop lung metastasis by ethical primary tumour endpoint (18–30 weeks of age) [24].

MIN has features consistent with human DCIS showing a solid growth pattern, cellular uniformity, even cell placement and central necrosis in some cases [11]. In a single field of view, many MIN lesions are visible in mouse mammary tissue, however only one-two DCIS lesions are visible in human tissue at the same magnification (200x, Fig 1). Invasive carcinoma has a clear loss of the normal orderly glandular architecture and increased architectural complexity in both mouse and human tissue. There were cases where it was difficult to distinguish MIN from early invasive carcinoma based on morphology alone. Therefore, we went on to investigate myoepithelial markers that could be utilised as a standardized method for scoring mammary gland tumour lesions going forward.

### Expression of myoepithelial markers throughout tumorigenesis

**Fig 2:**
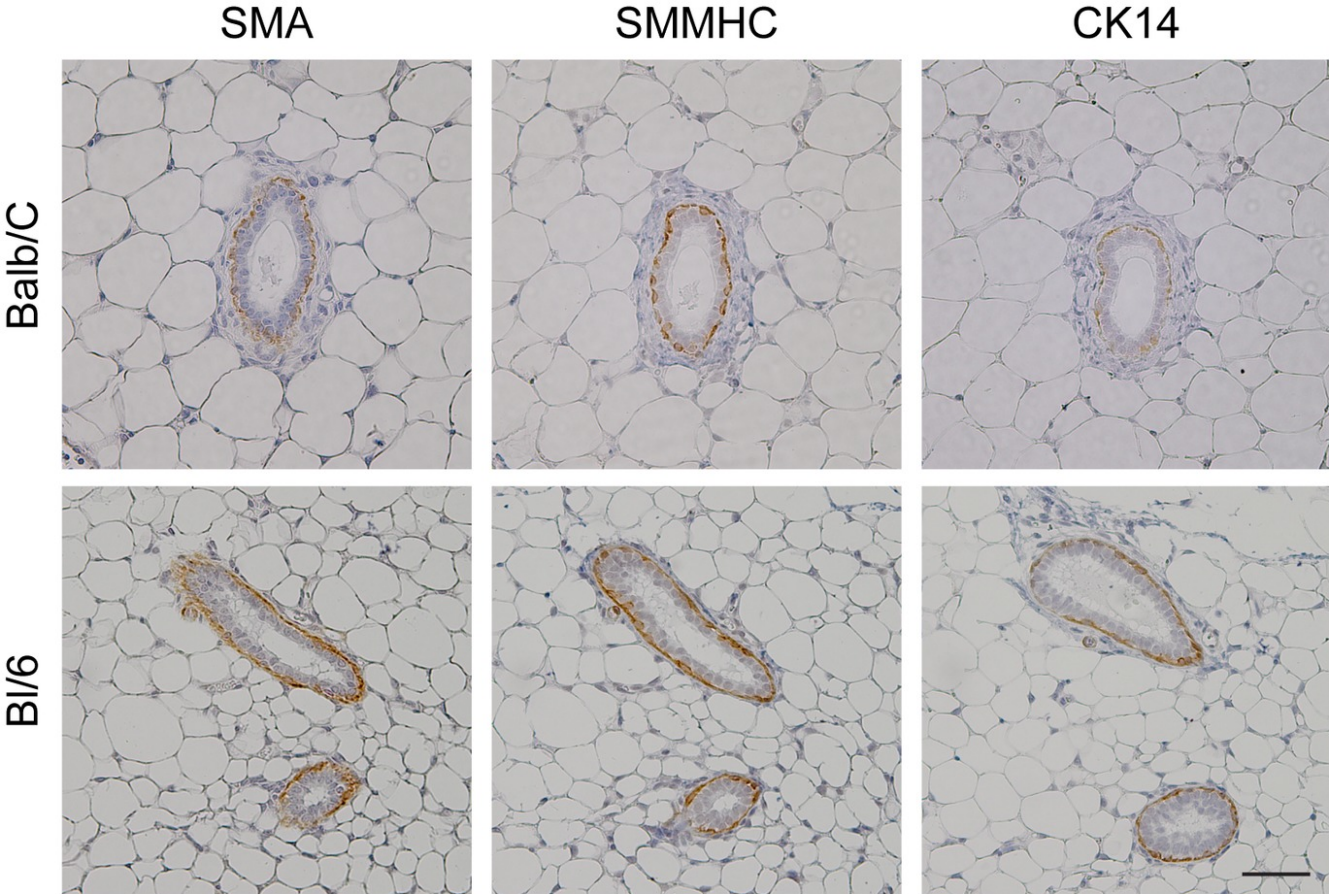
Myoepithelial markers on mouse mammary gland tissue. Sections of formalin-fixed, paraffin-embedded Balb/c and C57BL/6J mammary gland tissue were stained with a panel of myoepithelial markers, smooth muscle actin (SMA), smooth muscle myosin heavy chain (SMMHC) and cytokeratin 14 (CK14) and visualized with DAB (brown staining). All sections were counterstained with hematoxylin. Scale bar represents 50 μm.

We first assessed myoepithelial marker expression in normal ducts derived from adult C57BL/6J and Balb/c mammary glands. SMA and SMMHC highlighted the myoepithelial cell layer surrounding the luminal epithelial cells (Fig 2) in both models, whilst CK14 was strain specific, with clearer expression observed surrounding the ducts of C57BL/6J tissues. This indicates that SMA or SMMHC can be used as a myoepithelial marker in non-tumour mammary glands of these widely used mouse strains.

**Fig 3:**
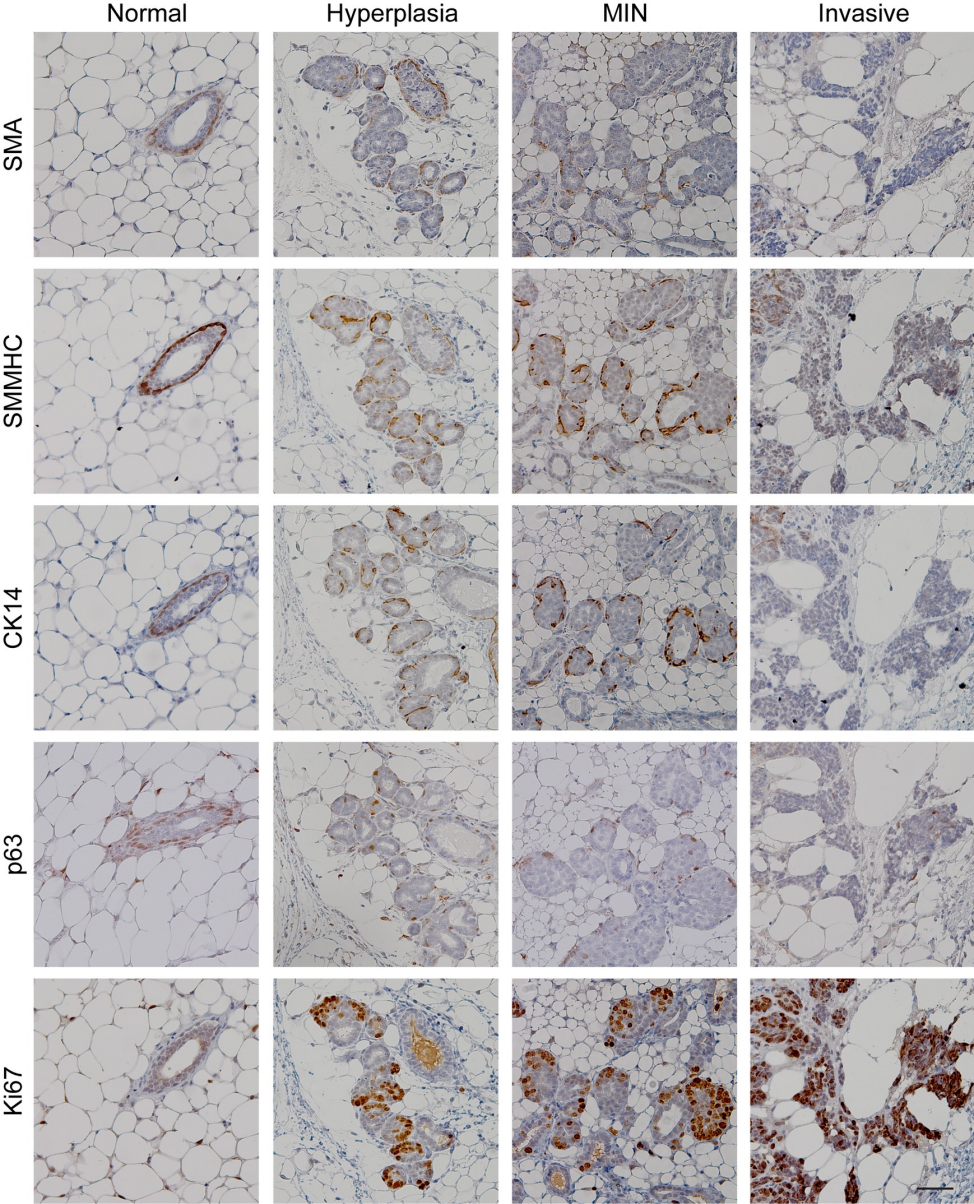
Identification of mouse myoepithelial markers in C57BL/6J MMTV-PyMT mammary gland tissue. Sections of formalin-fixed, paraffin-embedded C57BL/6J MMTV-PyMT mammary gland tissue were stained with Ki67 and a panel of myoepithelial markers and visualized with DAB (brown staining). All sections were counterstained with hematoxylin. Tissue sections containing normal ducts, hyperplastic, MIN and invasive regions (determined by a pathologist) were compared to determine the clearest myoepithelial markers for mouse tissue. Scale bar represents 50 μm.

To identify the optimal myoepithelial markers in tumorigenesis models, three mammary gland tissue areas from three C57BL/6J MMTV-PyMT positive female mice were examined. For normal, hyperplastic and MIN tissue from C57BL/6J MMTV-PyMT mice, antibodies against SMMHC and CK14 were superior for myoepithelial staining (Fig 3 and Table 1), with expression observed in 100% of mammary glands analysed from our laboratory. SMA and p63 exhibited myoepithelial expression in normal tissue, yet expression was substantially lost in hyperplasia and MIN compared to SMMHC and CK14 (Fig 3 and Table 1) with less than 25% of cells surrounding the lesions retaining expression. As expected, expression of myoepithelial markers was not detected in invasive areas of C57BL/6J MMTV-PyMT tumours.

**Table 1:** Expression of myoepithelial markers and Ki67 in C57BL/6J MMTV-PyMT mice.

	Normal	Hyperplasia	MIN	Invasive
**Average** number of ducts per field [0.3mm field diameter, 200x magnification] (range)^a^	**2** (1–3)	**17** (11–28)	**22** (19–24)	N/A
**Average** percentage of epithelial cells that express Ki67 (range)^a^	**7%** (1–17%)	**39%** (35–42%)	**55%** (50–58%)	**72%** (63–82%)
SMA^b^	Present	Present/ attenuated	Present, limited expression	Absent
SMMHC^b^	Present	Present	Present/ attenuated	Absent
CK14^b^	Present	Present	Present/ attenuated	Absent
p63^b^	Present	Present/ attenuated	Present, limited expression	Absent

^a^Range provided in brackets.

^b^Present: >75% of myoepithelial cells are positive. Attenuated: < 75% of myoepithelial cells are positive.

### Use of a proliferative marker to distinguish earliest lesions

**Fig 4:**
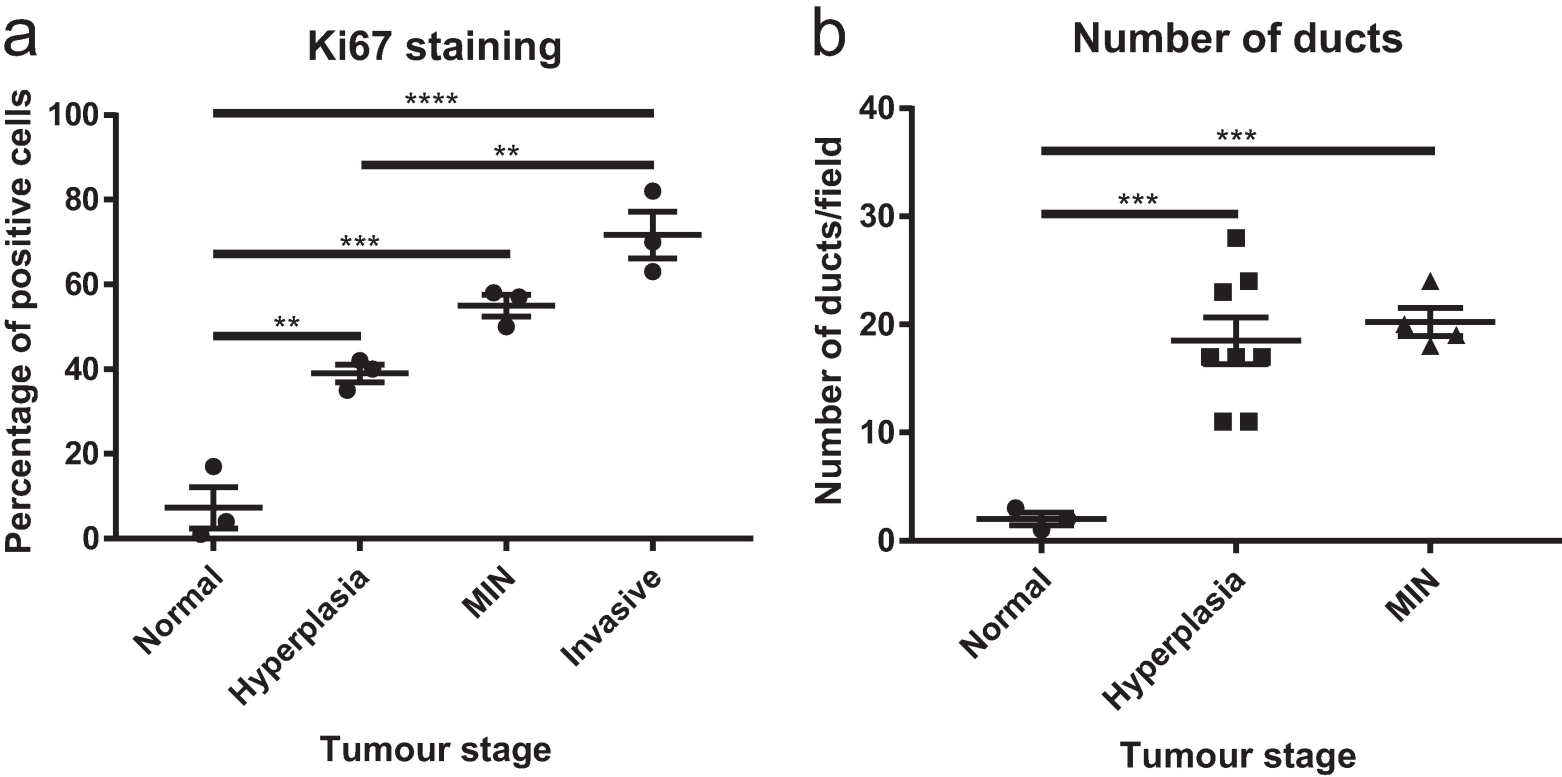
Ki67 and number of ducts per field are indicative of tumour stage. (A) The percentage of Ki67-positive cells were scored and averaged from three independent sections from three mice. (B) The number of ducts per microscopy field (0.3mm field diameter, 200x magnification) were counted with independent images from at least three mice. In invasive areas, ducts are not clearly distinguishable and were therefore not included in this analysis. **p<0.01, ***p<0.001, ****p<0.0001. *n* = 3. Error bars represent SEM.

In contrast to myoepithelial staining, Ki67 expression, which indicates an increase in proliferation, was expressed most abundantly in the invasive regions of tissues derived from the C57BL/6J MMTV-PyMT mice (Fig 3 and Table 1), as expected due to the high rate of growth of invasive tumour upon palpation in this model [24]. Importantly, we identified a notable difference of Ki67 expression between normal and hyperplasia, suggesting that an increase in proliferation is evident even at this early stage of tumorigenesis (Figs 3 and 4A). In our hands, greater than 30% of epithelial cells expressing Ki67 was indicative of a hyperplastic lesion (Figs 3 and 4A, Table 2). A difference in normal and hyperplastic lesions was also apparent in the number of ducts counted per field (0.3mm field diameter, 200x magnification, Fig 4B and Table 1), with greater than 13 ducts per field indicating hyperplasia (Table 2). Considering the difficulties in discerning normal mammary gland from regions of hyperplasia in this model using myoepithelial markers, these results suggest that Ki67 expression and number of ducts per field are distinguishing characteristics that are distinct between groups and could therefore be used to standardise scoring for these states in the C57BL/6J MMTV-PyMT model (Fig 4 and Table 2).

**Table 2:** Scoring matrix to distinguish different tumour stages in C57BL/6J MMTV-PyMT mice.

	Normal	Hyperplasia	MIN	Invasive
Appearance of lumen^a^	Empty lumen	Empty or slightly filled	Filled lumen	No lumen
Number of ducts per field (95% confidence interval; 0.3mm field diameter, 200x magnification)	0–4	13–24	16–24	N/A
Epithelial Ki67 positivity (95% confidence interval)	0–28%	30–48%	44–66%	48–96%
Expression of at least two myoepithelial markers (SMA, SMMHC, CK14, p63)^b^	Present	Present or attenuated	Present or attenuated	Absent

^a^As previously illustrated for such lesions by Lin et al. [14]

^b^Present: >75% of myoepithelial cells are positive. Attenuated: < 75% of myoepithelial cells are positive.

**Fig 5:**
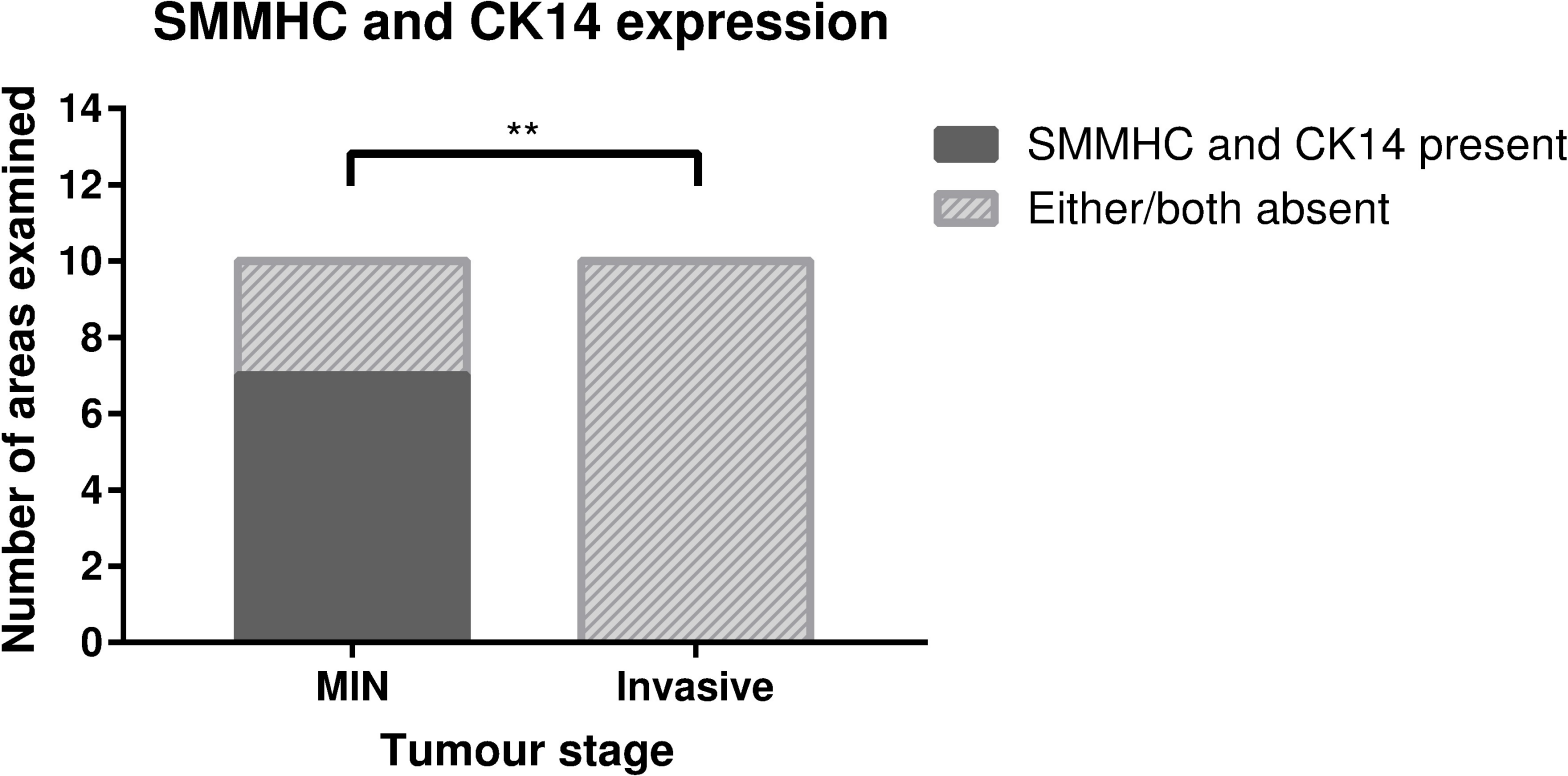
Scoring SMMHC and CK14 expression to distinguish MIN from invasive lesions in C57BL/6J MMTV-PyMT mice. The expression of SMMHC and CK14 in myoepithelial cells were scored and averaged from independent areas from five mice. Two-sided Fisher’s exact test, p = 0.0031 (**p<0.01) *n* = 5 mice.

The scoring matrix (Table 2) suggests the use of at least two myoepithelial markers, especially to distinguish MIN from invasive lesions in the C57BL/6J MMTV-PyMT model. To confirm this, statistical analysis of expression of SMMHC and CK14 in myoepithelial cells of MIN and invasive lesions was conducted (Fig 5). This identified that the expression of both SMMHC and CK14 classified MIN lesions, while the absence of one or both of these markers discriminated invasive lesions from MIN lesions.

## Discussion

Identifying robust myoepithelial markers to distinguish early stage MIN from invasive areas is imperative for the use of mouse mammary tumour models for early tumorigenesis studies. This study was conducted to identify myoepithelial markers that can be used to detect the presence of myoepithelial cells in normal mammary gland and early tumorigenic tissues.

Earlier studies with FVB [25] and C57BL/6J [26] mice reported SMA could be used to distinguish myoepithelial cells from luminal epithelial cells in normal ducts. Likewise, Lin et al. [14] determined SMA to be a suitable myoepithelial marker in the FVB-C3H/B6 MMTV-PyMT model. Further, in our previous study [22], we also identified SMA to be a suitable myoepithelial marker for both normal and MIN lesions in the C57BL/6J MMTV-PyMT model. While we found SMA to be suitable in this current study, after extensive characterisation we consider SMMHC and CK14 to be more specific myoepithelial markers in the C57BL/6J MMTV-PyMT model, especially for discriminating MIN lesions from invasive carcinoma. SMMHC and CK14 were expressed in a higher percentage of myoepithelial cells in all tissues examined and loss of expression was comparably less in MIN lesions compared to non-tumour bearing mice. Herein, we describe appropriate markers for the C57BL/6J MMTV-PyMT model, however, the MMTV-PyMT transgene is available on different backgrounds and we recommend that these markers are confirmed to be appropriate for each model before adoption.

It was clear from our evaluation of these myoepithelial markers, that MMTV-PyMT mouse MIN lesions do not have a prominent myoepithelial layer such as that seen generally surrounding human lesions, and that this layer is attenuated around the ducts in mouse mammary tissue. This was noted with low-grade MIN lesions demonstrating a dramatic reduction in SMA, SMMHC, CK14 and p63 expression compared to normal non-tumour bearing glands. In support of this finding, Lin et al. [14] also noted an incomplete layer of myoepithelial cells (as determined by SMA staining) around MIN stage lesions. This may suggest that myoepithelial cells alter their expression of ‘usual’ myoepithelial markers due to influence from the tumour cells and are therefore not detected by this staining. The alteration of markers in myoepithelial cells in early tumorigenesis is not limited to mouse models. Previously it has been noted that myoepithelial cells associated with DCIS lesions have reduced expression of multiple myoepithelial markers including SMMHC, p63 and calponin compared to normal myoepithelial cells [9, 10]. At the gene expression level, DCIS-associated myoepithelial cells lose a number of differentiation markers in comparison to normal myoepithelium [27, 28]. This has also been reported when evaluating the cell surface marker CD10, where loss has been documented at the DCIS stage [9, 29, 30], limiting the ability to immunopurify myoepithelial cells from fresh DCIS tissues.

Alternatively, the loss of myoepithelial markers in MIN lesions could also indicate the physical loss of myoepithelial cells during tumorigenesis–an event shortly preceding tumour cell invasion. A function of normal myoepithelial cells in mammary gland development and tumorigenesis is to limit epithelial cell growth and invasion. During the initial growth of the ductal structure, the proliferating edge of the terminal end buds is not surrounded by myoepithelial cells allowing cellular proliferation and migration during ductal elongation [25] and there are numerous reports linking the physical and molecular capacity of myoepithelial cells to block invasion. In the transgenic mouse model, it is possible that loss of myoepithelial cells occurs well before evidence of tumour cell invasion. Even the best myoepithelial markers identified in this study (SMMHC and CK14) had a reduction in cellular staining at the periphery of pathologist-identified MIN lesions. Therefore, we suggest that the presence of any myoepithelial markers may suggest MIN lesions, and the lack of myoepithelial marker expression indicates invasive areas in the C57BL/6J MMTV-PyMT model (Table 2). As is the case in human breast pathology, experienced pathologists do not generally rely on a single myoepithelial marker, to avoid the possibility of a false positive diagnosis of invasion due to the loss of expression of a sole myoepithelial cell marker. We suggest the use of at least two markers to provide additional rigor to the classification of lesions in these models.

Although SMMHC and CK14 distinguished MIN from invasive carcinoma, these markers could not always discriminate hyperplasia from normal ducts, with significant overlap within these groups. Ki67 antigen is expressed in proliferating cells during all phases of the cell cycle except G_0_, and is often used as a proliferative marker in tumorigenesis [31]. Our study revealed that Ki67 epithelial cell positivity is clearly enhanced in hyperplastic regions of C57BL/6J MMTV-PyMT mouse tissue compared to normal tissue. As hyperplasia is generally more subtle in mouse mammary glands where it is characterised by an increase in duct profiles, without necessarily observing intraluminal proliferation of ductal cells as seen in usual ductal hyperplasia in human breast tissue, we suggest that Ki67 staining is a useful marker to clearly distinguish normal (<28%) from hyperplastic ducts (30–48% epithelial positivity) in the C57BL/6J MMTV-PyMT mice (Table 2). Some hyperplastic ducts had decreased expression of myoepithelial markers, suggesting the layer is already disrupted at this very early stage of tumorigenesis. In line with this, investigations in patient-derived DCIS tissues has revealed that disruption of the myoepithelial boundary in breast cancer patients correlated with an increase in Ki67 staining [8]. The link between epithelial proliferation and myoepithelial loss in transgenic mouse models deserves further investigation.

Here, we have identified Ki67 and myoepithelial SMMHC and CK14 as robust markers for distinguishing normal epithelium, hyperplasia, MIN and invasive carcinoma in the C57BL/6J MMTV-PyMT model. Our data reveals that myoepithelial markers suitable for normal ducts are not always appropriate for studying early steps of tumorigenesis and that mammary tumour models need to be well characterised by individual laboratories to enable accurate prediction of the pre-invasive and invasive state.
